# Tracing the path of cancer initiation: the AA protein-based model for cancer genesis

**DOI:** 10.1186/s12885-018-4739-1

**Published:** 2018-08-17

**Authors:** Adouda Adjiri

**Affiliations:** Physics Department, Faculty of Sciences, Sétif-1 University, 19000 Sétif, Algeria

**Keywords:** Cancer hallmarks, Cancer-stem cells, Senescence, Adaptation, Inflammation, The switch from normalcy-to-malignancy, The AA protein-based model for cancer genesis

## Abstract

**Background:**

Cancer is a defiant disease which cure is still far from being attained besides the colossal efforts and financial means deployed towards that end. The continuing setbacks encountered with today’s arsenal of anti-cancer drugs and cancer therapy modalities; show the need for a radical approach in order to get to the root of the problem. And getting to the root of cancer initiation and development leads us to challenge the present dogmas surrounding the pathogenesis of this disease.

**Results:**

This comprehensive analysis brings to light the following points: (*i*) Cancer with its plethora of genetic and cellular symptoms could originate from one major event switching a cell from normalcy-to-malignancy; (*ii*) The switching event is postulated to involve a pathological breakup of a non-mutated protein, called here AA protein, resulting in the acquisition of new cellular functions present only in cancer cells; (*iii*) Following this event, DNA mutations begin to accumulate as secondary events to ensure perpetuity of cancer. Supporting arguments for this protein-based model come mainly from these observations: (*i*) The AA protein-based model reconciles together the clonal-and-stem cell theories into one inclusive model; (*ii*) The breakup of a normal protein could be behind the cancer-linked inflammation symptom; (*iii*) Cancer hallmarks are but adaptive traits, earned as a result of the switch from normalcy-to-malignancy.

**Conclusions:**

Adaptation of cancer cells to their microenvironment and to different anti-cancer drugs is deemed here as the ultimate cancer hallmark, that needs to be understood and controlled. This adaptive power of cancer cells parallels that of bacteria also known with their resistance to a large range of substances in nature and in the laboratory. Consequently, cancer development could be viewed as a backward walk on the line of Evolution. Finally this unprecedented analysis demystifies cancer and puts the finger on the core problem of malignancy while offering ideas for its control with the ultimate goal of leading to its cure.

## Background

Cancer is a complex disease that has defied scientists and clinicians over generations. Though the emergence of new diagnostic and treatment technologies has changed the face of cancer care today, death figures caused by this disease continue to be on the rise worldwide. Studies have predicted 23.6 million new cancer cases worldwide each year by 2030 [1]. The cancer challenge is largely due to the continuous trend of resistance to drugs; as cancer cells seem to mock all our efforts aimed at controlling their growth in order to repress tumor formation and spread. When numerous and innovative treatments are constantly met with resistance, it leads us as a consequence to question the fundamentals of this disease and seek to define the problem of malignancy more accurately. Therefore and besides its plethora of genetic and cellular symptoms, cancer remains one disease that should better be described as transformation. This transformation changes the cell’s fate switching it from normalcy-to-malignancy i.e. from controlled-to-uncontrolled growth. Deciphering the nature of this switch is needed in order to devise an efficient drug with long-lasting positive effects and lead finally to cancer cure.

The question is what to target in cancer in order to achieve a genuine recovery of cancer patients? If the drugs so far used to treat cancer did not result in cancer cure and eradication; the targets aimed by those drugs may not be the real cause of cancer [2]. Moreover, if DNA mutations and their derived mutated proteins cannot qualify as causal events in cancer [3]; what is their role and why do they accumulate by hundreds in cancer cells? Another important question is why cancer cells do not die under the heavy load of accumulated mutations and chromosome instability but thrive with their created genetic havoc? The answer to these questions is of a capital importance and will certainly lead to explain malignancy. Two important observations become obvious here: First cancer could be the result of a single switching event common to all forms of malignancies. Second, an in depth analysis of cancer hallmarks led to put the finger on the remarkable adaptive power of cancer cells. It is this adaptive property that made cancer cells resist PARP inhibitors by devising not one but three different strategies, as reported in literature [4, 5]. This is to list one example among a multitude of resistance cases to panoplies of anti-cancer drugs. Such a fascinating aspect of cancer cells to adapt is what makes them reprogram their metabolism according to whichever challenge is threatening their survival.

Tracing the path of cancer initiation hints us to suggest a model that could most likely explain malignancy. Cancer may come to existence if a normal protein, named here AA protein, is pathologically broken and that its resulting byproducts gain new functions by giving cancer cells unprecedented power for adaptation. The novelty of this model lies in the protein nature of the cause of cancer as opposed to DNA mutations occurring in a set of genes traditionally described as drivers of cancer. According to the model set forth here, accumulated mutations seen in cancer cells are considered as secondary events following the switch from normalcy-to-malignancy. Moreover their gene products could serve as tools necessary for perpetuating the malignant character down-on to future cancer-cell generations.

In this work we will first discuss the possibility that cancer could be initiated by a single event common to all malignancies. Second, the protein-based model for cancer genesis, termed here the AA model will be described. Third, arguments in favor of this model will be presented leading us as a consequence to review the notion of cancer stem-cells described in literature. Fourth, the powerful adaptive capacity of cancer cells compels us to make a parallel with bacteria which are known with their resistance to a very large range of toxic molecules from heavy metals to radiation all the way to antibiotics. The behavior of cancer cells may be seen as an attempt to regain primitive life features used by prokaryotic cells to survive. Indeed cancer survives as a single cell in its primary site and also in its metastatic site after detaching from the primary tumor. Finally, this in depth analysis could open unprecedented new venues in cancer research and lead to a comprehensive and genuine control of this disease and ultimately to its cure.

## Transformation could be initiated by a single event switching a cell from normalcy-to-malignancy

A cell is a universe of its own where each organelle function and each metabolic pathway, is interwoven with one another in a complex but remarkable manner leading to an amazingly organized unit forming the basis of life; i.e. the cell. Disorganizing this harmonious function without killing the cell but on the contrary giving it a property for unlimited cell division, as is the case in cancer, could only be achieved when the initiating and controlling event behind is shared, linking together mutational changes and watching over transformation as it progresses. Without this powerful control, rerouting any cell from mortal-to-immortal fate may be unviable because of the burden of genetic mutations and chromosome instability [6] tolerated only in cancerous but not in normal cells [3].

### Transformation is a coordinated process as it results in live cells capable of division

The most obvious and powerful evidence in favor of cancer being originated from a single switching event, common to all malignancies, resides in the fact that transformation results in live—as opposed to dead—cells which are capable of growth, division and also invasion and metastasis. Once the switch has occurred, cancer is already there and DNA mutations become essential in order to reprogram the metabolism of a normal cell and engender as a result a malignant character.

Numerous studies have shown that the mutations occurring in cancer cells affect two major categories of genes described as oncogenes (OCGs) and tumor suppressor genes (TSGs). All types of cancers so far described in literature refer to a proto-oncogene that has been activated and/or a tumor suppressor gene that has been inactivated [7]. Mutations in these genes determine cell cycle processes that control the tumor formation and development [8, 9]. The question asked here is: if cancer is orchestrated by one common transforming event, these major gene categories should be simultaneously affected. A short answer is provided by a survey conducted by Zhu K et al. who concluded that defects in these gene categories work jointly to engender cancer [10].

### Deregulation affects simultaneously both proto-oncogenes and tumor suppressor genes

In normal cells, proto-oncogenes code for proteins that send a signal to the nucleus, to stimulate cell division. These signaling proteins act in a series of steps known as signal transduction pathways. Oncogenes which are mutated versions of the proto-oncogenes activate the signaling pathway continuously, resulting in an increased production of factors that stimulate growth. The transforming properties are mediated in this case through gain-of-function mutations, shifting from highly regulated homeostatic signaling to an uncontrolled oncogenic situation [11]. The most studied oncogenes known to be altered in tumors are the receptor tyrosine kinase *EGFR* [12], *RAS* [13], *PI3K*/*AKT* [14] and *MEK*/*ERK* [15]. *MYC* is another pleiotropic transcription factor and a potent proto-oncogene reported to be frequently deregulated in human cancers, activating genetic programs that orchestrate biological processes to promote growth and proliferation [16].

Tumor suppressor genes on the other hand make proteins that normally inhibit cell growth and prevent tumor formation. *p53* as a potent tumor suppressor can trigger DNA repair processes and also induce the transcription of other tumor suppressors, such as *p21* and *p16*, and also initiate cell apoptosis [17, 18]. Mutations in tumor suppressor genes generally result in a loss-of-function of the resulting proteins which become unable to inhibit cell growth and prevent cancer. Moreover impairment of TSG functions, and unlike oncogenic events, requires the loss of both alleles.

Strikingly though certain genes can function as either oncogenes or tumor suppressor genes. A *p53* mutant has been shown to interact with *ETS2*, a member of the *ETS* family genes involved in diverse cellular pathways including apoptosis, angiogenesis, cell growth, adhesion, migration/invasion, the extracellular matrix, and other transcription factors [19]. Such interaction allows the *p53* mutant to hijack the ETS transcriptional pathways and control them for cancer promotion [20]. Another example involves *PTEN* loss/*AKT* activation pathway where a switch of p27 from a tumor suppressor to an oncogenic protein is seen and this was achieved through phosphorylation mediated nuclear-cytoplasmic translocation [21]. Moreover P53 and PTEN proteins both control cell death and proliferation and they are often expressed simultaneously in various types of tumors and jointly participate in the carcinogenesis of many malignancies [22]. The switch of such genes from a tumor-suppressive character to an oncogenic character may also argue in favor of cancer being orchestrated by the same controlling event. This modulation shows the remarkable flexibility of cancer cells reflecting their adaptive power to their microenvironment. Moreover, converting a tumor suppressor gene into an oncogene may translate into a more aggressive behavior of the cancers in which this occurs.

Furthermore, these observations show that inactivation of the tumor suppressor gene *PTEN* results in activation of the *AKT* kinase and inactivation of tumor suppressor gene *APC* results in constitutive activity of oncogenes such as *c-MYC* and *CTNNB1* [23–25], whereas, inactivation of the tumor suppressor gene *CDKN2A* results in activation of kinases such as CDK4, which bypass cell checkpoints [26]. Such dual action on tumor suppressor genes and proto-oncogenes could be facilitated only when the promoting agent and/or mechanism is shared. Such co-operative action, deactivating tumor suppressors and enhancing proto-oncogenes strongly argues in favor of cancer being driven by the same cellular modification playing a causal role. Moreover TSG silencing has been suggested as an early initiating event in the process of oncogenesis. *CDKN2A* silencing was registered in the mammary tissue of women at high risk for breast cancer [27]. Other studies have demonstrated a premalignant zone surrounding a primary breast tumor where TSGs were found silenced [28, 29]. Moreover *PTEN* is shown to be the most frequent tumor suppressor lost in human cancers [30].

Following this line of thinking it is reasonable to expect an increase of anti-apoptotic and anti-senescence activities concomitant with a decrease of pro-apoptotic and pro-senescence activities in cancer cells. For a successful transformation, survival and proliferation of cancer cells, these actions should be kept under tight control otherwise any attempt to deregulate a normal cell through an oncogenic activation would be aborted by a suppressive action of a TSG.

In conclusion simultaneity of events, activating oncogenes while deactivating tumor suppressor genes; means there is coordination, and if there is coordination there is control, and if there is control; chances are that this control is exercised by the same agent.

## The AA protein-based model for cancer genesis

The complexity of cancer as a disease compels us to review this pathology in its context of Evolution but also to question present dogmas surrounding tumor genesis. This is crucial in order to unlock the enigma that is shaping cancer and get out of the circle of resistance/recurrence seen in clinics today. For this, a thorough analysis of cancer hallmarks coupled with a global vision of all its aspects as seen through the window of Evolution; led as a consequence to model cancer initiation and development as most likely being caused by a pathological breakup of a normal protein, as opposed to DNA mutations which involve the formation of abnormal and probably not-optimally functioning proteins.

The rationale behind this protein-based model for cancer genesis took shape after following these steps: (*i*) a mutated protein (or two mutated proteins) has (have) been ruled out as a cause of cancer because that would take us back to DNA events and DNA mutations which already have been ruled out as a probable cause of cancer [3]. What remains then was to look into normal proteins for the cause of cancer; (*ii*) but how a normal protein could be behind a disease like cancer? If this protein is cut into two pieces; it would fit and fulfill the “two-hit” hypothesis for cancer genesis; (*iii*) how a single cut in any protein would fulfill the two hit (i.e. two actions) hypothesis? Only if the engendered protein-pieces can generate new functions that are not found in non-malignant cells; (*iv*) the question asked then is: which protein when cut would do the job and explain to us what’s happening in cancer cells? Here a thorough protein sequence analysis led to put the finger on the AA protein as the unique candidate protein capable of explaining –at least theoretically– the molecular mechanism behind the deregulation seen in cancer cells. The predicted actions of the protein pieces A1 and A2 engendered from the breakup of AA protein and described below are based on their relative sequences.

**Fig. 1:**
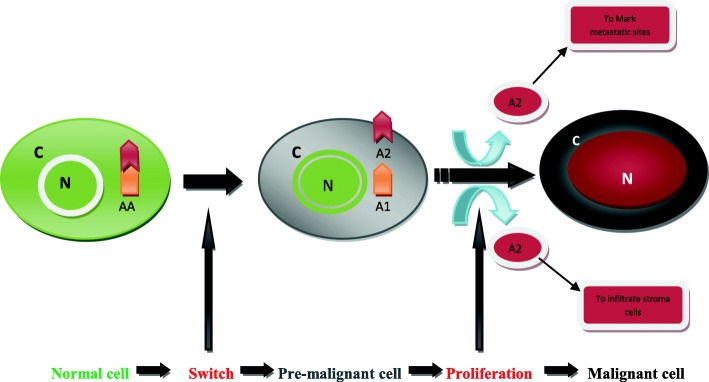
Simplified AA protein-based model for cancer genesis: In a normal cell AA protein forms one unit. In pre-malignant cells a pathological breakup of AA generates two entities A1 and A2, marking the switch from normalcy-to-malignancy. Both A1 & A2 acquire each a new activity not present in non-transformed cells. A2 infiltrates the stroma cells and also marks sites for metastases to form. C: cytoplasm; N: nucleus

Traditionally, the path for cancer initiation and development has been linked to the occurrence of a series of DNA mutations affecting sets of genes qualified as drivers or passengers depending on their contribution to the tumorigenic process [31, 32]. This view where DNA mutations were put at the center of cancer initiation and development has been recently challenged [3]. The model described here puts instead a protein at the center of cancer initiation and development, highlighting the novelty of this model. The protein here is not a modified protein resulting from a DNA mutational event but a non-mutated and functional protein called for now AA protein. Based on its sequence, cancer initiation and progression is therefore postulated to result from the pathological breakup of this normal AA protein giving rise to two entities A1 and A2. Each entity is postulated to acquire a new cellular activity equipping cancer cells with new features giving them a selective advantage over normal cells (Fig. 1). This pathological breakup of a functional protein constitutes the switch event which takes a cell from normalcy-to-malignancy.

While the resulting A1 entity may localize to the cytoplasm, A2 entity is thought to travel and mark sites for future metastases, as it could keep a copy of itself on the cell membrane to likely serve as a new transduction signal in cancer cells. Generations of cells are needed to form a tumor and because of the protein nature behind the cause of cancer, cancerous cell devise DNA mutations as secondary events to ensure their perpetuity. The formation of DNA mutations in selected genes allows those growth advantages earned at the switch, to become fixed on the DNA so that the malignant character can continue in the event A1 protein is lost or destabilized. Unlike a protein entity, fixed mutations on the DNA can be more faithfully transmitted to cancer daughter-cells. More importantly though stress levels may change throughout the life of a tumor and if stress drops below transformational levels; cells may become unable to continue to break up AA proteins and therefore unable to generate A1 and A2 entities vital for cancer existence (Fig. 1).

In summary cancer may begin with the pathological breakup of a functional and normal protein giving rise to two active moieties with new functions that are not present in non-cancerous cells. These formed protein entities cannot assure an indefinite propagation required to serve the immortal character of cancer cells. Therefore DNA mutations arise as secondary events to ensure perpetuity of cancer, and their accumulation during cancer development – as opposed to cancer initiation – may be proportional to the degree of malignancy achieved by each cancerous cell. A full malignant cell is that cell, once released from the primary tumor, is capable of initiating new tumors and form metastases. A less or non-malignant cell is that cell that is unable to start new tumors or form metastases due to instability and/or early loss of AA byproducts and consequently do not harbor enough cellular modifications that would give them a full malignant character. Moreover metastatic sites may not meet the environmental conditions in which the breakup of the AA protein could continue.

The question remains however in the great number of mutations registered in cancer cells: do cancer cells need all those mutations to function? Are there wanted and unwanted-but-tolerated mutations in cancer cells? Could the wanted mutations include those seen in the frequently mutated genes (traditionally called driver mutations)? And could the unwanted mutations (traditionally called passenger mutations) be simply tolerated, or could their protein-product be recycled to serve cancer growth needs? Future investigations will likely shed light on this point and answer those questions.

## Inflammation creates cancer and cancer creates inflammation

### High stress levels could help cells escape senescence and trigger transformation

To create cancer, stress is needed and as Einstein once stated “nothing happens, until something moves”. In parallel, no disease manifests itself until some sort of stress is applied on a given cell or organ. For stress to create cancer; it has to reach transformational levels at least transiently, leading here to the pathological breakup of a normal and functional protein. This model postulates that the switching event marked by the breakup of AA protein may occur in pre-senescent cells because it is known that once cells have completed their senescence program, they do not revert to reenter the cell cycle; a feature generally used to define senescent cells [33]. Although it is not impossible to imagine that the breakup of a normal protein could in itself switch a senesced cell into a cancerous cell. Moreover, the genetic and epigenetic landscape of pre-senescing (or senesced) cells could serve as a fertile cellular ground, giving an important thrust to the first formed cancerous cell to survive and make its first round of cell division without being subject to apoptotic death or immune attacks and before resorting to DNA mutations to reinforce and delegate such protective measures. It is therefore vital for a cell that has escaped senescence to resist apoptosis and immune system defensive power as they forge their way towards immortality. Senescent cells are resistant to apoptosis, a property shared by cancer cells, and cancer cells have been observed to arise from among senescing cells [34]. In the case of telomere dysfunction induced senescence, Beauséjour CM et al. [35] have shown that the senescence response is a reversible event that is primarily maintained by p53 protein and that the dominant and second barrier to the unlimited growth of human cells is provided by p16, a protein controlling entry into senescence and which expression and function have also been demonstrated to be independent of telomere status [36].

To reach transformational levels, stress has to be chronic and chronic stress is behind chronic inflammation. Senescent cells through the secretion of senescence–associated secretory phenotype or SASP factors are demonstrated to directly or indirectly promote inflammation (Reviewed in [37]). Senescent cells create through inflammation a tissue microenvironment accommodating cancer development and may also promote its initiation [37]. Moreover, chronic inflammation has been reported to be an important contributor to major age-related diseases [38]. On the other hand, secretion of SASP has been described as a plastic phenotype and proteins secreted may vary with cell types and, to some extent, with the stimulus that induced the senescence response [37]. Therefore this plasticity may influence the pathway of progression of cells escaping senescence, giving rise as a consequence to genetically and morphologically heterogeneous cancer cells. An important investigation showed that epigenetic factors can activate pro-inflammatory reactions underlying activation of SASP as demonstrated in the case of the methyltransferase mixed-lineage leukemia 1 (MLL1). During cellular senescence, the MLL1 protein activates the expression of proliferation-related cell cycle genes, causing hyperreplicative stress that triggers the DNA damage response. This leads as a consequence to the activation of the NF-κB pro-inflammatory signaling pathway that drives SASP gene expression [39].

Though their biological activities are complex, SASP factors can stimulate new blood vessel formation and induce an epithelial-to-mesenchymal transition in senescent fibroblasts. Cancer cells arising from senescence may carry these important features which are known as cancer hallmarks [40]. Moreover the SASP comprises proteases of the matrix metalloproteinase and serine protease family, which normally facilitate tissue repair through degradation of collagen and regulate the activity of other SASP factors. Another senescence feature that may be exploited by cancer cells is the secretion of high levels of MMPs and cancer cells emerging from a senescence milieu may carry this feature as well. Over-expression of MMPs associated with metastasis has been described in different cancers including lung, breast and colon cancer [41–43]. Moreover, MMPs are often over-expressed in tumors and especially in the tumor stroma [44].

Therefore the negative effects of an enduring inflammation could lead to the breakup of a normal protein, as a way of escaping senescence, giving therefore cells unprecedented growth and survival advantages. Another advantage of a senescence milieu is that inflammation and SASP effects could serve as a barrier protecting nascent cancer cells from being eliminated or from triggering unwanted defensive mechanisms. Moreover, the switch event involving the breakup of AA protein creates an additional stress, tilting the balance of senescence from beneficial to detrimental and instead of promoting optimal healing, it rather creates cancer.

### A breakup of a normal protein could be behind the cancer hallmark of tumor-promoting inflammation

The link between chronic inflammation and the rise of cancer may be explained by the pathological breakup of AA protein. This highly stressful event can exacerbate an ongoing inflammation and contribute as a consequence to its endurance. Fueling inflammation may allow first cancer cells to establish themselves before any suspicious micro-tumor could be detected. During this critical period, cancer cells may generate more A1 and A2 byproducts. Accumulation of such byproducts may determine how fast and how aggressive a tumor might become. Moreover tumors have been described as a state of never healing wounds [45] which could be explained here by the stress response engendered by the pathological breakup of an otherwise normal protein, creating as a consequence a state of chronic inflammation.

On the other hand, in normal wound healing, fibroblasts, inflammatory cells and mesenchymal stem cells infiltrate the wound and remodel the microenvironment therefore organizing angiogenesis and cell proliferation to repair the tissue [46]. While wound healing is a transient response, the switch from normalcy-to-malignancy maintains inflammation, preventing therefore the wound from healing. Found also in the stroma are mediators of the innate immune system i.e. macrophages, neutrophils and mast cells [47, 48]. These cells may be reeducated by cancer cells if infiltrated by A2 entity, to serve cancer growth, facilitate invasion and metastases formation.

Also of importance, elevated levels of Reactive Oxygen Species (ROS) may lead to macromolecular damages including proteins, lipids, and nucleic acids which are involved in important mechanisms responsible for cellular senescence, aging and in the development of several age-associated diseases [49]. ROS can also induce senescence via telomere-dependent and-independent mechanisms involving non-repaired single or double-strand DNA breaks [50, 51]. This observation shows a link between ROS formation, generation of DNA damage, and senescence. Such a link reinforces the idea of cancer emerging from a senescence milieu. Moreover it points to the fact that the most important effects resulting from DNA damage are genomic instability and mutations which are important cancer hallmarks.

## Control over autophagy is needed to safeguard A1 and A2 byproducts and take over normal adaptive capacities of cells

Autophagy is a highly regulated cellular process vital to cell homeostasis by which cytoplasmic material is brought to lysosomes for degradation. This ensures continuous renewal of proteome and organelles in normal conditions of cellular life [52, 53]. Autophagy is also crucial to mediate cellular adaptation to environmental changes as well as to respond to intra-and-extracellular stressors [54]. Moreover, studies have shown that autophagy is involved in different aspects of anticancer immune-surveillance where the immune system constantly eliminates potentially tumorigenic cells before they become malignant [Reviewed in 55]. In contrast to normal cells, autophagy is shown to be important for the survival of tumor cells that can have high levels of basal autophagy and be constitutively dependent on autophagy for survival [56–58]. Autophagy is also found to be induced in hypoxic tumor regions; conferring cancer cells with a survival advantage [56].

Therefore and in order for cancer to emerge, grow and metastasize, it has to overcome autophagic and immune barriers. There are several reasons for which cancer cells would opt to take early control over autophagy: (*i*) Autophagy mediates potent anti-inflammatory effects [59] and this role would play against the interest of cancer cells which need inflammatory conditions in order to continue and induce the escape from senescence, as described in the AA model; (*ii*) Cancer cells must protect A1 and A2 entities from degradation knowing that autophagy is suggested to be involved in the degradation of oncogenic proteins including mutant P53 [60–62]; (*iii*) Autophagy in immune cells has been shown to be implicated in several steps of both innate and adaptive responses [55], and cancer cells have to control both these processes to ensure their survival and propagation; (*iv*) Advanced human tumors show an increased autophagic flux, in correlation with an invasive/metastatic phenotype, high nuclear grade, and poor disease outcome [63, 64] and such high flux could only be possible when cancer cells have full control over autophagy. Moreover, in established tumors, metabolic stress induces autophagy as cancer cells seek an alternative source of energy and metabolites [65–68].

**Fig. 2:**
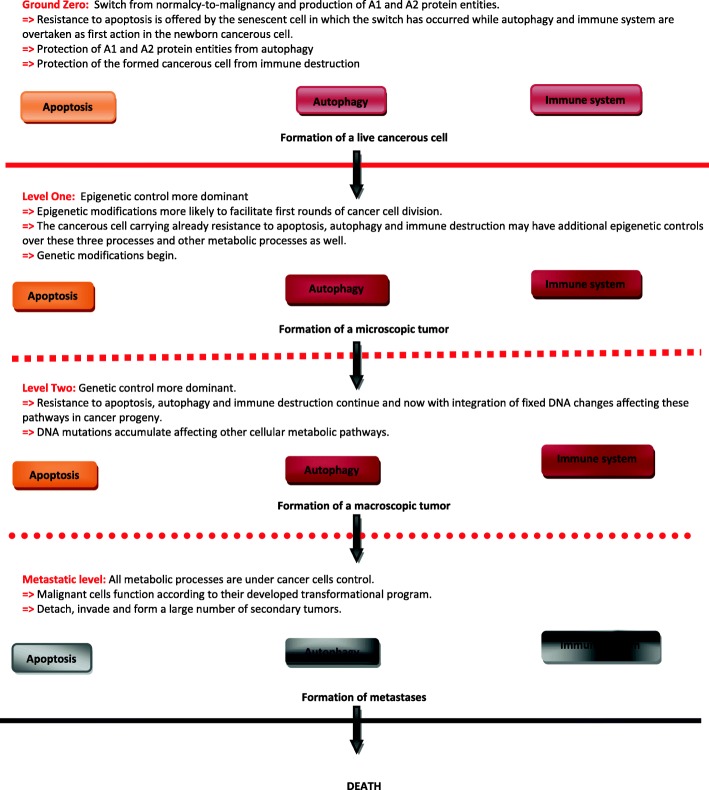
Control of death processes by cancer cells: a synchronous action over apoptosis, autophagy and immune system is essential to protect cancerous cells from their birth till metastases formation. Cancer cells die off when the hosting patient succumbs under the burden of metastases

While autophagy and immune surveillance are linked, which both constitute imminent danger to cancer cell survival; it follows that both processes must be controlled early in the course of tumor genesis (Fig. 2). Therefore any successful tumorigenic event must go side-by-side with control over autophagy and immune system surveillance; otherwise any attempt to create cancer would be halted and cancer cells eliminated before becoming malignant. In the AA model, safeguarding A1 and A2 from autophagic destruction is pivotal for cancer genesis and could implicate an early control over not only autophagy but also the immune system through a dual control. Figure 2 depicts a summary of cancer cells control over autophagy and immune system but also over apoptosis; all three processes constitute a threat to the survival of cancer cells. A synchronized control over these three processes is deemed capital for cancer initiation and development. Epigenetic interventions, suggested here to be offered by a senescence program, could also help cancer cells exercise their controlling power over autophagy. Cruickshanks HA et al. have shown that gains and losses of methylation in replication-induced senescence to be qualitatively similar to those in cancer and that this methylation landscape is retained when cells bypass senescence. Therefore such DNA methylome of senescent cells might promote malignancy [69].

Control over autophagy (and the immune system) is needed throughout the life of cancer cells from initiation to metastases formation. While safeguarding A1 and A2 protein entities is a good reason for cancer cells to take control over autophagy, they also may equally want to take control over autophagy as an adaptive process of normal cells to different stressors [54]. Cancer cells want particularly to control this normal adaptive process and hijack it to serve adaptive needs of cancer cells instead; ensuring therefore cancer proliferation, resistance to immune system attacks and more importantly resistance to anti-cancer drugs.

## Arguments in favor of the AA protein-based model for cancer genesis

Arguments in favor of the protein-based model described above can be summarized in four major points: (*i*) the protein-based model for cancer genesis redefines cancer stem-cells and (*ii*) reconciles present theories of clonal evolution versus cancer stem-cell hypotheses; (*iii*) formation of metastases argues in favor of cancer being governed by an event that has initiated at the primary tumor site showing transformation as a coordinated event; (*vi*) major cancer hallmarks highlight senescent cells features and show cancer cells’ unprecedented adaptive power.

### The AA protein-based model redefines cancer stem-cells

Normal stem cells are described as a type of cells distinguished with three unique properties: (*i*) they can self-renew to perpetuate and maintain a pool of undifferentiated stem cells; (*ii*) they can differentiate in multiple lineages and; (*iii*) they can maintain a balance between self-renewal and differentiation. Three types of stem-cells are known: embryonic stem-cells which give rise to all the different cells in the adult organs; germinal stem-cells responsible for reproduction; and somatic stem-cells present in different tissues [70].

Cancer stem-cells (CSC) as described in literature, behave like normal stem-cells with their capacity to self-renew giving rise to different progeny and use general signaling pathways including the *Hedghog*, *Notch* and *Wnt* signaling pathways [71]. Other shared properties include active telomerase expression, activation of anti-apoptotic pathway, increased membrane transporter activity and ability to migrate [72]. Cancer stem-cells differ however from normal stem-cells with their tumorigenic capacity that enables them to form tumors when transplanted into animals; a feature lacking in normal stem-cells that are unable to form tumors.

In the laboratory, specific markers are used to identify CSC populations and most currently identified CSC markers are derived from normal embryonic or adult stem-cell surface markers (Reviewed in [73]). Since these CSC markers are shared with normal embryonic or adult stem-cells they obviously are not specific of cancer cells. And if these markers are not specific of cancer cells; can we still rely on them to identify cancer stem-cells? If the answer is negative then the question of how to distinguish a cancer stem-cell from a normal stem-cell, and for that matter a cancerous cell from a normal cell, remains open. How to recognize cancer stem-cells and clear the ambiguity surrounding their identification may all be settled in the AA protein-based model describing cancer genesis. This model allows us therefore to redefine cancer stem-cells as those cells harboring A1 and A2 byproducts resulting from the pathological breakup of the AA protein. The presence of these byproducts in cancer cells while absent in normal cells is what defines a cancer stem-cell, according to the AA model, because the difference is clear-cut; distinguishing a pathological cell from a normal cell. Each cancer cell producing and harboring these byproducts is therefore a cancer stem-cell which conserves its capacity for clonal evolution while showing a heterogeneous phenotype linked to the protein nature of A1 and A2 byproducts which transmission through cell generations could be hampered or lost.

If that is what’s really happening in cancer stem-cells, why then markers of stemness of normal cells are expressed by cancer cells? What normal stem-cells markers could do is give cancer cells full advantages normally owned only by normal stem-cells such as telomerase expression and protection advantages with resistance to apoptosis. Expression of normal stem-cells markers by cancer cells does not make them gain cancer-stemness property per se because this characteristic as modeled here is earned from the time of the breakup of the AA protein. Moreover, it has been shown that the activation of embryonic stem-cell (ESC)-like gene expression in adult cells is considered to provide the ability of self-renewal to cancer stem-cells [74]. In addition, cancer and embryonic cells share other features such as similar morphology, increased proliferation rate, ability to invade tissues, evasion of immune destruction and secretion of angiogenic factors. On the other hand, it has been shown that Cripto-1 (TDGF1: Teratocarcinoma-derived growth factor 1) expression is shared by both embryonic cells and cancer cells. Cripto1 has been demonstrated to promote cancer cell migration, proliferation, epithelial-mesenchymal transition (EMT), angiogenesis and its expression is increased several-fold in human colon, gastric, pancreatic, lung, and breast carcinomas ([75] and reviewed in [73]). Therefore and reaching a stage where cancer cells express normal stem-cell markers, may indicate their readiness to invade and metastasize as those stemness features give them further advantages needed to achieve metastatic objectives.

While cancer cells are known to revert to a stem-like type of cells, markers used to define their stemness should be unique and specific to cancer cells as being the pathological cells. In this regard, data from the JS Morisson lab may be in support of the AA model described here. If markers of stemness defining normal stem-cells are also those defining stemness in cancer cells, transplantation of such cancer stem-cells should always result in the formation of tumors when engrafted into experimental animals. This wasn’t the case as demonstrated by the same research group where a total of 85 stem-cell markers failed to distinguish tumorigenic from non-tumorigenic melanoma cells [76–78]. Therefore what defines stemness in normal cells does not extrapolate to define stemness in cancer cells. What defines stemness in cancer cell could well be related to the presence of AA breakup byproducts which are deemed absent in all types of normal cells including normal stem-cells.

### The AA protein-based model reconciles the clonal-and-cancer stem-cell theories

Currently two models describe the development of tumors; the clonal evolution model and the cancer-stem cell model [79]. In the clonal evolution model all cells within a tumor, which have accumulated epigenetic and genetic changes, can become invasive, cause metastases, and contribute to resistance to therapies and ultimately to recurrence of the disease. The cancer stem-cell model suggests on the other side that cancer stem-cells, which form a subset of the tumor, are the ones responsible for tumor initiation, progression and recurrence. According to this model cancer stem-cells are directly responsible of resistance to therapy and metastases formation [79].

In the light of this work and if the etiology of cancer is of protein nature as opposed to DNA mutations in a given set of genes, which ultimately will give rise to mutated proteins, the whole story changes and consequently our perception of this notorious disease also changes. This paradigm shift is maybe what is needed to move a step forward and make cancer a curable disease. Therefore, if A1 entity is present in the cytoplasm, its transmission to daughter cells can parallel that of mitochondria distribution following somatic cell divisions, in opposition to the transmission of mutated forms of genes fixed on the DNA. In yeast, when unequal distribution of mitochondria occurs; it results in the petite phenotype.

The presence of a low A1 copy number or loss in some daughter cells may explain why some cells of a tumor fail to grow and/or to continue to grow into tumors when transplanted into animal models. The most important entity however in this two-hit model is the presence of A2 fraction, predicted to play an adaptive role as a new transduction signal in cancer cells. Therefore the fraction of cancer cells harboring enough copies of A1 needed to make a tumor and A2 needed to adapt is maybe what the cancer stem-cell model calls as cancer stem-cells. Moreover one should be aware that transplanting cancer cells into animal models may not replicate the microenvironment of tumor cells at their primary site, known to be important to sustain tumor growth, invasion and metastasis formation. In addition, transplantation experiments in animal models may not recreate sufficient stressful and inflammatory conditions to sustain a malignant phenotype.

On the other hand, the frequency of cancer stem-cells, as defined in the traditional models of tumor formation; have been demonstrated to vary dramatically between tumor types and also between tumors of the same origin [80]. These differences show the higher plasticity displayed by cancer stem-cells [81] which may be explained in the AA protein-based model as probably related to: (*i*) A1 and A2 production linked to variations of stress levels directly responsible of their creation; (*ii*) A1 copy number, stability and uneven transmission to cancer daughter-cells.

In conclusion all cancer cells bearing A1 and A2 entities which have directly resulted from the pathological breakup of a normal AA protein are called cancer-stem cells. These A1- and-A2-bearing cancer stem-cells have a clonal growth however with variable degrees of malignancy due to the protein nature of the cancer-causing entities invoked here and also to their related transmission to cancer daughter-cells and how faithful this transmission is ensured. Therefore there will always be within a primary tumor a fraction of cancer cells that have attained full malignancy, making them capable of initiating new tumors on their own relying perhaps at this stage much more on the expression of their accumulated mutated proteins, and non-malignant cells unable to initiate tumors on their own or continue to grow when engrafted. Those cells that have attained full malignancy make a fraction within a tumor as described in the traditional cancer stem-cell model. The clonal development of such cancer stem-cells will always give rise to a mixed population of cells forming a malignantly-heterogeneous tumor. Therefore both models; the clonal and the stem-cell models, reconcile when cancer is projected to be caused by protein entities but not initiated or caused by DNA mutations postulated here to rise as secondary events following the switch from normalcy-to-malignancy that is engendered by the AA protein breakup.

### The AA protein-based model supports metastases formation as a coordinated event governed by a common event initiating at the primary site

Metastases formation is a complex biological process comprising a cascade of events summarized in eight steps as flows: When cancerous cells reach the stage of metastases formation they breach the basement membrane barrier; dissociate from the tumor mass; invade neighboring tissue; intravasate into pre-existing and newly formed blood and lymph vessels; transported through vessels; extravasate from vessels; disseminate, at a secondary anatomical site; and develop into secondary tumors (Reviewed in [82]). The formation of pre-metastatic niche has recently been added as a step (0) marking the sites for new tumors to be formed [83]. Moreover metastases are described as clonal events [84] and that the tendency of cancer cells to metastasize is largely determined by the genes expressed in the mass of the primary tumor [85]. These observations are in accordance with the model projected here. In addition the number of secondary tumors and their simultaneous appearance, point to the likely hood of these events being governed not only by the initial event which occurred at the primary tumor site, but also supports the idea of transformation as a coordinated event. Therefore clonality and stemness are projected to be both present in cancer cells at the metastatic sites resulting in faster and simultaneous appearance of these secondary tumors.

It is worth noting that A2 fraction could not be restrained to cancer cells only and to marking sites for secondary tumor formation; it can also integrate within vicinal normal cells present in the stroma and use them as a guide and shield during cancer cell movement. This could greatly facilitate the travel of cancer cells, knowing that they can face various threats during the metastatic process at any of the steps enumerated above. It has been demonstrated that the normal cells residing in the immediate vicinity of the tumor, the tumor stroma, play an essential role in tumorigenesis, both at early and late stages of tumor progression [86, 87]. Therefore if normal cells in the stroma integrate A2 fraction, they could be subdued to play a supportive role when competent cancer cells begin their move during invasion and progress towards metastases formation.

Moreover, cancer cells in movement have to overcome a major barrier imposed by anoikis, a cell death process induced by inappropriate or loss of cell adhesion, which normally plays a role in metastasis-suppression [88]. Being shielded by stromal cells bearing A2 entity could easily help these cells overcome the threats imposed by anoikis. It has been demonstrated that Tumor-Associated Macrophages (TAMs) facilitate tumor cell intravasation into vessels [89]. In the light of this work and as stated before, A2 could mark sites for future secondary tumors but could also cover additional adaptive needs at the secondary sites. Conditions at secondary sites could be more challenging in the sense that the survival of a cancerous cell as a single cell is a highly risky business. At the metastatic sites, A2 could infiltrate into tissue cells building-up a niche suitable for the roaming single cancer cells to land and multiply into colonies. A2 alone cannot make cells cancerous without the help of A1 function. A2 may thus subdue the normal cells present in the secondary sites; preventing them from destroying or reacting to the presence of an abnormal cell and here a cancerous cell.

On the other hand cancer cells which have reached the stage of metastases formation, have major cellular metabolic pathways under their control. When pro-senescence and pro-apoptotic genes have been inactivated and major oncogenes produced according to the needs of malignant cells; metastasizing cancer cells waste therefore no time for forming new colonies making them as a consequence grow simultaneously. Unlike the situation at the primary site where heterogeneous and mixed population of cancer cells are present; cell populations at a given metastatic site are expected to be more homogeneous. However, different tumor-metastases, started by different malignant cancer cells deriving from the primary site, are expected to be heterogeneous (inter-metastases heterogeneity). Finally the observations presented above are supportive of the AA model and in good accordance with the original hypothesis of “seed and soil” first described by Paget in 1889 [90] and reviewed by Fidler [91].

### Major cancer hallmarks highlight senescent cells features and show cancer cells adaptive power

**Fig. 3:**
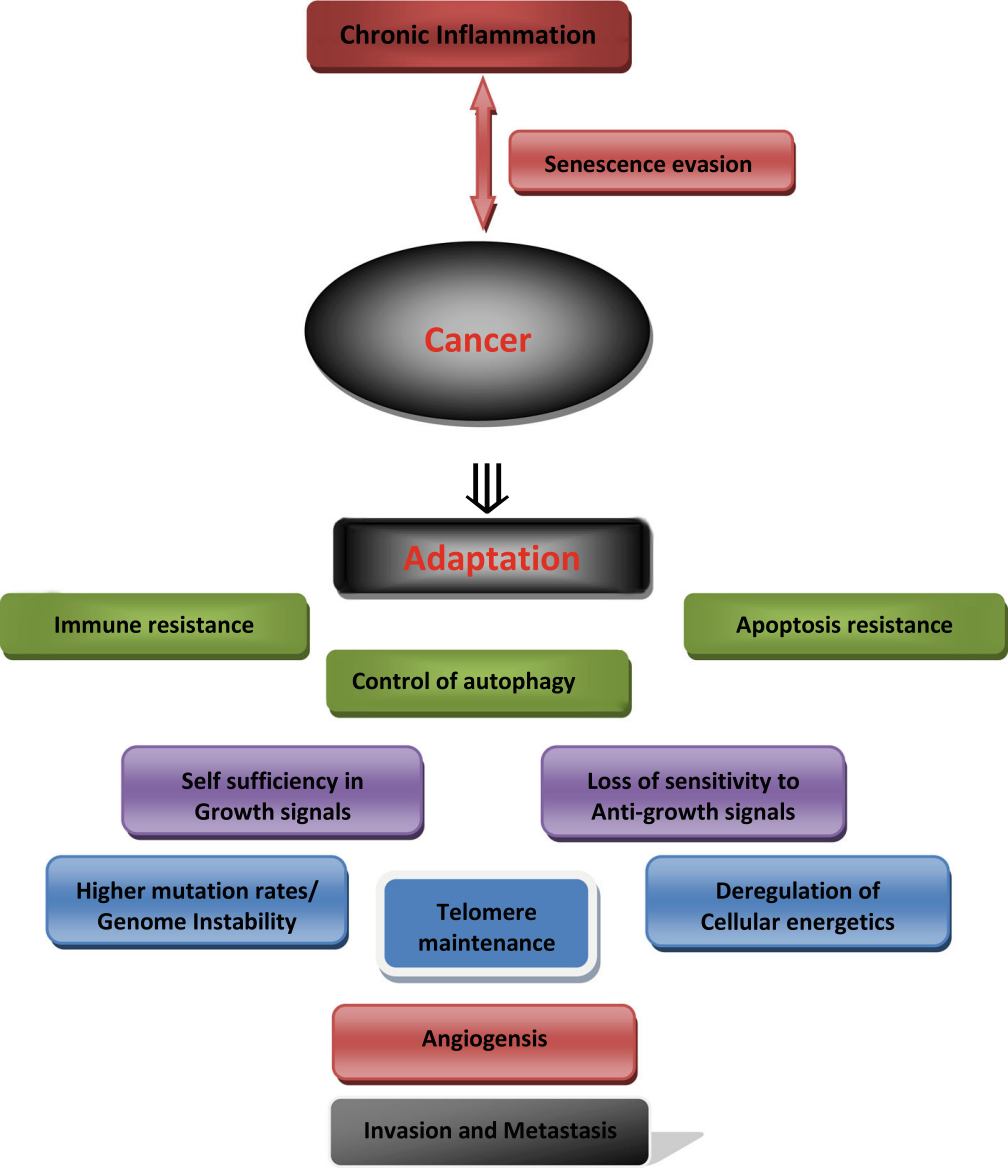
The hallmarks of cancer as viewed in the AA protein-based model for cancer genesis: evasion from senescence is coupled with the breakup of AA protein into A1 and A2 entities leading to the formation of a cancerous cell. Inflammation is at the head of cancer hallmarks fueled by the breakup of AA protein (dark brown). Immortality of cancer cells is linked to the acquisition of a highly adaptive capacity, leading therefore to first control death processes i.e. apoptosis and immune destruction, in addition to a control over autophagy, added here as a cancer hallmark (green). This three-dimensional control continues throughout the life of cancer cells and may involve different mechanisms. Loss of sensitivity to anti-growth signals and self-sufficiency in growth signals follow as early traits earned at the birth of cancer (purple). The latter facilitate the reprogramming of normal cellular metabolism to create malignancy through accumulation of epigenetic-genetic changes and chromosome instability in parallel to deregulation of cellular energetics (blue). Multiple rounds of cell division need telomere maintenance (blue light). Once the tumor becomes large enough it creates new blood vessels to gain access to oxygen/nutrients (pink), and prepares for invasion and metastases formation (black)

The hallmarks of cancer have been described by Hanahan D and Weinberg RA [40] and while evading senescence is not listed as a cancer hallmark; senescence landscape is postulated to be the basis for the emergence of cancer cells according to the AA model and supportive literature arguments. Cancer hallmarks highlight as a consequence senescent cells features and could emerge because of favorable epigenetic and genetic landscape of senescing cells and their microenvironment. In addition to the observations outlined in section four, striking similarities between senescent cells and cancer cells have also been described in literature [69]. Figure 3 summarizes cancer hallmarks as adapted to the AA protein-based model for cancer genesis.

First of all, promoting inflammation (hallmark 1) may be fulfilled by the breakup of AA protein creating stress and fuelling more inflammation. The obvious scenario is that the originally present inflammation helps cancer cells escape senescence while in return the stress caused by the breakup of a normal protein fuels inflammation and thus makes the wound a “non-healable wound”, allowing the establishment of first-born cancer cells. Second, from the ten cancer hallmarks described by Hanahan and Weinberg, resistance to apoptosis (hallmark 2) appears as a pre-existing condition offered by senescent cells which themselves are resistant to apoptosis. Initial survival is the most critical step in cancer development therefore minimal conditions are required and could only be provided by senescent cells in terms of their resistance to apoptosis. Later on the path of tumorigenesis, cancer cells can devise additional pathways to resist apoptosis in order for tumor cells to successfully metastasize to distant sites. Resistance to apoptosis is certainly needed during tissue invasion, transport through blood and lymph vessels and after extravasation at distant anatomical sites. Therefore the switch from normalcy-to-malignancy must give cancer cells additional means to resist apoptosis during their evolution toward metastases formation. Moreover cancer hallmarks while offering cancer cells survival advantages show their highly adaptive power. Cancer cells may overcome the need for proliferative signals (hallmark 3) or the need to respond to growth arrest signals (hallmark 4) in one token by using A2 as a new transduction signal unique to cancer cells. It is known that senescent cells are readily less sensitive to external growth factors which may facilitate the role of A2 as a new but pathological growth signal. This observation enforces the idea of cancer emerging not from pre-senescing cells but rather from senesced cells and it is the breakup of AA protein which makes them reinter the cell cycle to produce cancer.

The other remaining cancer hallmarks are also seen as clear adaptive responses to intra-and-extra-cellular conditions: introducing mutations and chromosome instability (hallmark 5), is an event through which cancer cells delegate survival properties to their progeny where mostly tumor suppressor genes are inactivated and oncogenes activated. The growth of cancer cells into a tumor requires building blocks and suitable energy forms (hallmark 6) where malignant cells choose anaerobic over aerobic degradation of glucose which is an undisputed adaptive feature offering cancer cells survival means. When cancer cells are faced with short telomeres they adapt and reactivate their telomerase (hallmark 7) and when this telomerase activity is neutralized by anti-telomerase drugs, cancer cells again adapt and recur to alternative ways (ALT mechanism) to keep their telomeres intact and prevent a telomere-induced senescence response in cancer progeny. Therefore the AA model suggests that reactivation of telomerase is not what renders cancer cells immortal but rather their earned highly adaptive power is what drives them immortal. When the tumor has reached a given size, nutrients and oxygen cannot reach all parts of the tumor, therefore cancer cells adapt by creating new blood vessels for sustenance which can also serve as a venue for future invasion and metastasis formation (hallmarks 8 & 9). Hypoxic conditions promote not only sustained angiogenesis but can also induce and select for an invasive and metastatic phenotype [92]. The escape from immune surveillance (hallmark 10) as an important cancer hallmarks will be treated separately in the following section.

Finally, it is inevitable to conclude that all cancer hallmarks enumerated above are but adaptive features earned by cancer cells which have switched from normal and regulated-growth to transformed and unregulated-growth.

## Control over the immune system could be an early event earned at the switch from normalcy-to-malignancy

Dissecting further cancer hallmarks leads us to ask, which feature is more important for cancer cells to earn early on during the process of tumorigenesis to ensure a selective and a long-lasting survival advantage. And what feature is more important in cancer development than resisting destruction by the immune system at different levels; from initiation, to development, invasion and metastases formation? Would cancerous cells wait to create DNA mutations down the road in order to carry out this important hallmark and escape immune attacks? It is unlikely so because this could jeopardize first rounds of cell division when it is still easy for the immune system to clear few cancer cells, as compared to a mass of cells forming a visible tumor and much more when multiple metastases have mushroomed all over the body.

Therefore avoiding immune destruction is regarded as the most important cancer hallmark that may not be left to be dealt with down the road in future generations as the tumor accumulates DNA mutations. According to the AA model, immune resistance could be earned at the switch from normalcy-to-malignancy. The strongest argument for this assumption is the fact that cancer cells, after having established themselves at the primary site, and when ready to propagate, mine their way through lymph and blood vessels both of which should constitute great danger for cancer cells survival due to the presence of diverse immune killer cells. Moreover, when cancer cells reach lymph nodes and colonize them fearlessly; that in itself shows the power of malignant cells to defy human immune capabilities. Two scenarios are possible here: (*i*) either cancer cells are themselves rendered invisible to immune cells; or (*ii*) the immune system has been somehow reeducated to accept cancer cells as normal and non-dangerous cells. However, one possibility may not exclude the other.

While the AA model postulates cancer as arising from senescent cells; it is known that senescent cells are normally cleared by immune cells through the positive role played by SASP and their intervention in attracting immune system to clear both pre-malignant and established tumor cells by phagocytosis or cytotoxic-mediated killing as demonstrated in liver cancer for example [93]. If the immune system has the capability to clear pre-malignant and established tumors; how then cancer would come to exist, persist and thrive at the detriment of the organ and organism hosting it? If indeed cancer cells survive immune destruction, they may do so only if they have integrated this property in their cells to become invisible to all types of immune cell surveillance or have reeducated the immune system to not react to cancer cells. Could A2 entity postulated to be present on cancer cell surface serve as an epitope to signal for “self” instead of “non-self” and deter immune cells? Future investigations will be able to answer this question.

Moreover, cells which have succeeded to evade senescence and became cancerous may well have acquired the ability to not only escape immune attack at their birth place but also resist immune attack as long as they continue to proliferate. The decline of the immune system with age is not enough to argue for cancer resistance to immune system [94]; this is because cancer also develops in younger individuals where the immune capacities are not weakened as in the elderly. Moreover, without advising such an early strategy, every rising cancerous cell would have a high probability of being eliminated by various mechanisms of the immune system. Moreover different triggers may, at any step of cancer evolution and movement, be turned on at the detriment of cancer cells.

In conclusion, resistance to immune system could very likely be earned at the time of the switch from normalcy-to-malignancy. Moreover and if immune system resistance is not dealt with at the time of the switch, metastases formation could be aborted and deemed as unsuccessful events. The control that cancer cells may exercise on the immune system is expected to be limited to accommodate cancer cells. The fact that cancer cells use other cells as a shield during their movement, and the fact that secondary tumor sites need to be fashioned; means that the immune system is not a totally corrupt system in cancer. Total corruption of human immune system would be incompatible with life and what we see in cancer development is something more like a state of confusion than total corruption of immune functions.

## Adaptation as the ultimate cancer hallmark and the parallel with prokaryotic cells

Following this analysis it becomes obvious that when cancer switches a cell’s fate from regulated-to-unregulated state, the success of such a detrimental change could happen only through the acquisition of unprecedented adaptive power earned by cancer cells. This adaptive power challenged scientists and clinicians for decades and the more venues we try to limit cancer proliferation, the more resistance we seem to encounter. It leads therefore to suggest adaptation as the most important cancer hallmark under the umbrella of which cancer hallmarks described in literature are sheltered.

This adaptive power makes eukaryotic cells which have become cancerous behave in a manner similar to prokaryotic cells. Bacteria, as primitive forms of cellular life, are resistant to almost everything in nature including; heavy metals, high salt concentrations, high temperatures, multiple antibiotics, and much more they resist ionizing radiation. In parallel cancer cells have resisted all our attempts to kill them through chemotherapy or control their growth through targeted-and-radiation therapies. Cancer resistance was registered in response to a myriad of drugs and drug combinations as well.

While adapting to their microenvironment, mimicry emerged as a trait shaping cancer cells. Mimicry could be seen as a long-term survival strategy ensuring cancer cells growth, invasion and metastases. This feature facilitates the works of cancer cells and thwarts off attempts of immune cells and apoptotic interventions to exercise their neutralizing effects. Mimicry of wound healing may help cancer cells escape senescence by keeping stress levels on; needed for the establishment of the first cancerous cells. Mimicry of development may allow deviation of tissues’ resources in favor of tumor growth. Mimicry of normal stem-cells adds further growth and proliferative advantages such as the reactivation of telomerase activities. Acquisition of embryonic features could shut down unwanted pathways in favor of pathways needed for cancer cells propagation. Mimicry of embryogenesis also helps cancer cells in their transition from epithelial-to-mesenchymal cells (EMT) and following propagation the reverse process, i.e. mesenchymal-to-epithelial transition (MET) could help in establishing secondary tumors similar to the primary one [95, 96]. This plasticity, switching from one property to another; clearly shows how cancer cells can adapt and what strategy to choose in order to survive and continue to grow.

More striking though, faster amoeboid-like forms of migration are seen in tumor cell invasion and migration and these forms do not require proteolytic ECM remodeling [97]. Wolf K et al. have shown, in vitro and in vivo experiments, that cells can switch from mesenchymal-to-amoeboid migration and that this switch makes the cells independent of proteases and enables them to continue to invade in the presence of protease inhibitors [98]; just another example of adaptive behavior of cancer cells highlighted here by their resistance to anti-protease drugs. Therefore, the highly adaptive capability of cancer cells, including their resistance to our arsenal of anti-cancer components and multiple therapeutic modalities, should be recognized as the ultimate hallmark of cancer and targeted in a comprehensive manner.

More importantly though it looks like the more aggressively cancer cells are treated, the more adaptive they become. A recent study by Lewis K, Shan Y on prokaryotic cells, has shown that bacteria use tolerance and resistance as two mechanisms to avoid the killing effect of antibiotics and that resistance mechanisms such as destruction of a drug or modification of its target allow bacteria to grow in the presence of antibiotics [99]. Another work performed by Levin-Reisman I et al. has shown that tolerance of antibiotics leads to resistance and that both tolerance and resistance involve the acquisition of mutational changes. Moreover bacteria may use quiescence when antibiotics are around in highest concentrations (tolerance) or gain active biochemical resistance (resistance) [100]. A similar behavior may be occurring in cancer cells in a sense that when overwhelmed with toxic compounds of chemotherapy may go into quiescence to later resume their growth when the treatment stops leading as a consequence to cancer relapse.

## Cancer development could be seen as a failed attempt to revert evolution

Striking similarities between bacterial cells and cancer cells behavior are evident mainly when we consider these two manifest traits: (*i*) bacterial cells divide at faster rates compared to eukaryotic cells, singular or multi-cellular organisms, while cancer cells divide at faster rates compared to their normal counterparts; (*ii*) accumulation of mutations in bacteria allows adaptation to their environment such as resisting toxins or antibiotics, while cancer cells devise similar attributes to resist anti-cancer drugs. These observations may in one hand reinforce the idea that cancer could not possibly have been selected for by evolutionary forces [2] as such behavior is less perfect when seen on the scale of Evolution. On the other hand, multi-cellular organisms including humans have evolved out of the mutational era and the need to adapt faster to environmental changes in order to survive. Humans in particular out-evolved other multi-cellular organisms in the sense that they developed consciousness which helps them fashion their environment and survive without the need to mutate in order to accommodate themselves to their environment. However mutations continue to occur in humans but at a healthy level to allow variation. The mutation rate of stable genomes is estimated to be 10^− 10^/bp per cell generation [101].

Generation time of a bacterium is estimated between 20 and 30 min and that of a eukaryotic single yeast cell is around 100 min, while normal mammalian cell cycle takes 7.5–8.5 h. Shorter renewal times of a cell population have been shown to be associated with an elevated tumor incidence in human and rodent tissues [102]. Shortening generation time in cancer cells along with accumulation of high levels of DNA mutations; may be seen as a backward walk on the line of Evolution. Maurice Tubiana [103] observed that the non-binding of cancer cells to the mechanisms governing normal growth is one of the main characteristics of malignancy and that the degree of such unrestraint varies widely among human tumors even when they belong to the same histological type [103]. According to the same author a high proliferative rate appears to be the indicator of tumor malignancy where proliferation rate is higher in metastases than in the primary tumor, suggesting that the spread of cancer is initiated by cells belonging to rapidly dividing sub-clones. Moreover the author observed that a high proliferation rate is associated with a disturbance of cell-to-cell interactions and of cell contact inhibition [103]. This physical separation of malignant cells from their primary tumor shows yet another parallel with bacteria, living as single cells and growing to form tumor colonies. While mimicking such a behavior, cancer cells single themselves out of the primary tumor and demonstrate their capacity to live as independent cells when forming metastatic colonies. And while cancer cells survive and proliferate following separation, normal tissue cells survive and proliferate only when anchored to a solid substrate.

Moreover, it has been reported that invasive cancer cells can migrate either as single cells or collectively in the form of files, clusters, or sheets [97, 104]. This could be yet another but remarkable trait cancer cells may share with bacterial cells. Different bacterial genera are known to form different associations with *Streptococci* forming chains and *Staphylococci* grape-like clusters. While *Streptococci* divide along a single cellular axis and form chains, *Staphylococci* divide along multiple axes allowing them to form clusters. Orientation during cell division is a mechanism known to regulate cell fates helping to restrict tumor formation. Stem cells are known with their ability to generate both self-renewing and differentiating daughter cells, and this is possible because of the mechanism of asymmetric cell division. He Z et al. have recently demonstrated that suppression of *BRCA1* function alters the growth, phenotype and polarity of progeny cells. In in vitro experiment, the authors have revealed a new role for BRCA1 protein on mechanisms that regulate the cell division axis in proliferating, non-transformed human mammary epithelial cells [105].

On the other hand, accumulation of higher levels of mutations and chromosome instability in cancer cells entices one to describe cancer as a life-costing attempt to run a eukaryotic cell metabolism according to a prokaryotic model. While trans-differentiation of white fat cells to brown fat cells [106] may be evidence that de-differentiation is possible; this phenomenon may be very limited and strictly kept under control in order to continue to push forward the wheel of Evolution. And going forward, Evolution has integrated lower forms of life for the service of higher forms of life. Bacteria have become part of eukaryotic cells under the form of mitochondria and also part of animals’ intestine flora. Single eukaryotic cells serve multi-cellular organisms and help at least humans make their bread. Therefore, the lack of positive gains in cancer development leads to deduce that de-differentiation (if cancer is seen as an attempt to de-differentiate) that is not in the line of Evolution may be deemed to failure. When Evolution opts to select for an outcome, it protects it and allows its propagation throughout eons. Therefore bacteria which have subsisted for this long period of time did so successfully because: (*i*) they have been selected for by evolutionary forces and (*ii*) these evolutionary forces have selected in bacteria the power to adapt in order to survive for eons and continue to defy clinicians with their resistance to antibiotics. Cancer cells as eukaryotic cells which have learned how to adapt; kill and shorten lives of inflicted individuals, making cancer unlikely to be selected for by Evolution [2].

## Conclusions

This in depth analysis sheds unprecedented new light on cancer initiation and development. Today, advances in research technology coupled with accumulation of an impressive amount of data, make the moment ripe to begin to put together the pieces of the cancer puzzle. This work is first in its nature to suggest cancer initiation as being most likely caused by a protein breakup that switches the fate of a cell from normalcy-to-malignancy and that following this switch, DNA mutations begin to accumulate as secondary events in cancer cells. The model presented here is a two-hit model where an AA protein is broken to generate A1 and A2 byproducts, allowing the acquisition by cancer cells of new activities not present in their normal counterparts. Together these entities permit the fulfillment of the deregulation condition of cancer in which oncogenes are amplified and tumor suppressor genes inactivated.

Cancer could be the result of a single event switching the fate of a cell from normalcy-to-malignancy. The fact that a tumor suppressor gene can sometimes be inactivated through loss-of-function mutation and some other times transformed into an oncogene through a gain-of-function mutation; tells about three properties of cancer cells functioning: (*i*) their ability to fine-tune their cellular metabolism; (*ii*) this fine-tuning could be possible only when this manipulation is orchestrated by the same controlling agent; (*iii*) this fine-tuning highlights the power of cancer cells to adapt to different environmental circumstances. The AA model shows the malignant character of cancer cells as being directly linked to the acquisition of a highly adaptive power allowing cancer cells to resist intrinsic defensive mechanisms and extrinsic therapeutic interventions. Consequently all emanating cancer hallmarks are but traits showing powerful adaptive actions of cancer cells to their environment including the presence of therapeutic drugs.

Senescence could offer the appropriate conditions to cancer initiation and establishment. Studies have shown that when the senescence-promoting process does not result in actual senescence, can lead to a trail of genetically unstable cells, which potentially can contribute to tumor creation [107]. Escaping senescence could therefore be the unique way when cancer cells seek immortality [2]. Cancer cells want neither to age nor to die and this is maybe the reason for which they reactivate embryonic pathways and stem-cell features and inactivate death programs such as apoptosis and immune killing strategies. Moreover, senescence is considered as a general response in normal cells to various types of cellular damages [108]. And what would be more stressful in a cell than the breakup of one of its normal protein? While such an event exacerbates stress responses of senescent cells, it gives birth to cancer and allows the first cancer cells to establish themselves.

The AA protein-based model for cancer genesis redefine cancer stem-cells as those cells harboring the byproducts A1 and A2, directly resulting from AA breakup. While the AA model offers a unifying explanation for the clonal-and-stem-cell theories for cancer development, it suggests that DNA mutations in cancer are generated as secondary events following the breakup of AA protein, the event defining the switch from normalcy-to-malignancy. Through these DNA mutations, and given the protein nature of the cause of cancer, primary cancer cells delegate survival and metastatic properties to their progeny.

The observation in clinics of acute forms of transformation could also argue in favor of cancer being initiated by a single cellular change. The single change here is not a particular translocation because translocations are DNA events and are different in different hematological malignancies. In this case and given the natural speed of blood cells renewal, an acute malignant phenotype results as a consequence. This acute phenotype could also be explained by short cuts taken by a given translocation powerful enough to shunt transformational events and thus shorten the time for malignancy to become manifest. In this case inhibiting the overly expressed molecules resulting from translocation puts these cancer cells in serious jeopardy. However and as long as these blood cells continue to harbor or produce the entities responsible of the switch from a normal to a malignant character, recurrence becomes all but an expected outcome as seen in clinics today. In addition viruses-associated cancers may not depart from the rule of a switch from normalcy-to-malignancy as described in this model. What these viruses may do is bring with them an activity that could facilitate the works of A1 and/or A2 entities. It has been reported that protein viruses involved in tumor genesis of infected cells act and interact with telomerase [109].

When cancer is seen through the window of Evolution, it could be regarded as a backward walk on the line of evolution. This observation emanates from two major properties of cancer cells: (*i*) their fast growth reducing the time generally needed for non-transformed cells to grow; (*ii*) the accumulation of a great number of mutations through which they seem to tolerate and resist our therapeutic compounds and ionizing radiation. Behavioral similarities between cancer cells and bacteria lead us to see cancer resistance from a different angle. Under aggressive treatments caused by chemo-and-radiation therapies and if cancer cells become overwhelmed by stress, they may choose dormancy and quiescence until the level of cellular stress becomes manageable, for cancer cells to resume growth and result as a consequence in cancer relapse. Regarded from this angle; cancer becomes a matter for a eukaryotic cell to acquire highly adaptive features for survival mimicking bacterial life-style. Therefore, neutralizing this adaptive power will most likely result in the control of cancer-cell growth and their subsequent elimination. Moreover when silencing this adaptive mechanism, the existing targeted-therapy drugs are expected to give positive and better lasting results when the associated-risks of resistance through adaptation are blocked.

Finally, cancer treatment does not call for war nor does it need toxic weaponry; cancer rather needs understanding in order to well define the parameters shaping its initiation, growth, invasion and metastases formation. And this study offers valuable clues demystifying cancer while paving the road to its potential cure. Identifying and targeting the cause of cancer is needed to cure cancer [2], and what needs to be targeted more than the entity or entities responsible of the highly adaptive power of cancer cells? Targeting A2 (in concert with A1) will likely halt cancer growth allowing the body’s defense system to take over and lead effectively to their elimination. If A1 entity is permanently lost in the progeny; A2 is expected to remain as part of the membrane in metastasizing cells but also in cells in the stroma and the cells forming the niche. When A2 is neutralized; the immune system is no longer confused, will regain its power and role in eliminating cancer cells; just as Evolution selected it to accomplish this role and protect us from cancer.

The following step in this endeavor is to isolate A1 and A2 entities in all types of cancer cells and characterize their predicted new biochemical activities. Failure to prove the AA model in clinics is strongly doubtful but in the event that happens; someone has to come with a better idea than the breakup of a normal protein to explain cancer. Nevertheless, if there is one positive result coming out of this study; it is the adaptive trait of cancer cells that needs to be understood and targeted in order to put a limit to cancer resistance and lead patients to full recovery and lasting cure. The next few years will certainly witness a dramatic progress in cancer care and management.

## Abbreviations

AKT: Serine/threonine kinase 1
ALT: Alternative lengthening of telomeres
APC: Adenomatous polyposis coli/WNT signaling pathway regulator
BRCA1: Breast cancer 1
CDK4: Cyclin dependent kinase 4
CDKN2A: Cyclin dependent kinase inhibitor 2A
Cripto-1: TDGF1: Teratocarcinoma-derived growth factor 1
CSC: Cancer stem-cell
CTNNB1: Catenin beta 1
ECM: Extracellular matrix
EGFR: Epidermal growth factor receptor
EMT: Epithelial-to-mesenchymal transition
ESC: Embryonic stem-cell
ETS2: E26 transformation-specific proto-oncogene 2 trancription factor
MEK/ERK: Mitogen-activated protein kinase/extracellular signal–regulated kinase
MET: Mesenchymal-to-epithelial transition
MLL1: Methyltransferase mixed-lineage leukemia 1
MMP: Matrix metalloproteinase
MYC: Myelocytomatosis proto-oncogene
NF-κB: Nuclear factor kappa B
OCG: Oncogene
p16: Protein 16
p21: Protein 21
p53: Protein 53
PARP: Poly-(ADP-ribose) polymerase
PI3K: Phosphatidylinositol-4,5-bisphosphate 3-kinase
PTEN: Phosphatase and tensin homolog
RAS: RAS (Rat Sarcoma) proto-oncogene, GTPase
ROS: Reactive oxygen species
SASP: Secretion of senescence–associated secretory phenotype
TAM: Tumor-Associated Macrophages
TSG: Tumor-suppressor gene
Wnt: (wingless) Wnt signaling pathway
